# Comparing Jaffe and Enzymatic Methods for Creatinine Measurement at Various Icterus Levels and Their Impacts on Liver Transplant Allocation

**DOI:** 10.1155/2023/9804533

**Published:** 2023-10-12

**Authors:** Neda Soleimani, Sima Dehghani, Mohammad Hossein Anbardar, Sahand Mohammadzadeh, Elaheh Amirinezhad Fard, Atefeh Zare Sheibani, Mohammad Javad Esmaeili, Marsa Ebrahimi

**Affiliations:** ^1^Department of Pathology, Shiraz Medical School, Shiraz University of Medical Sciences, Shiraz, Iran; ^2^Department of Pathology, Shiraz Transplant Center, Abu Ali Sina Hospital, Shiraz University of Medical Sciences, Shiraz, Iran; ^3^Medical Immunology, Department of Pathology, Shiraz Transplant Center, Abu Ali Sina Hospital, Shiraz University of Medical Sciences, Shiraz, Iran; ^4^Medical Laboratory, Department of Pathology, Shiraz Transplant Center, Abu Ali Sina Hospital, Shiraz University of Medical Sciences, Shiraz, Iran

## Abstract

The Model for End-Stage Liver Disease (MELD) scoring system is used to prioritize liver transplantations and assess disease severity. This includes the international normalized ratio (INR), creatinine, and total bilirubin. Since there are several ways to measure creatinine, MELD scores can produce inconsistent results. The objectives of this study were to define a valid cut-off for bilirubin interference in creatinine measurement and to assess the effects of various icteric levels on creatinine measurement and liver transplant allocation. A total of 400 serum samples were categorized into four groups based on their icteric indices and total bilirubin levels, including non-, mild, moderate, and severe icteric samples. Both chemical Jaffe and enzymatic techniques were used to determine the creatinine levels in all four groups, and the findings were compared. In parallel, serum samples from 83 liver transplant candidate patients were divided into three groups depending on their bilirubin levels and then similarly evaluated and interpreted. The MELD scores were then computed for each group and compared. In icteric samples, the enzymatic method produced higher results for the creatinine concentrations than the Jaffe method did, and the mean creatinine difference rose from 0.08 in nonicteric group to 1.95 in groups with severe icterus. In addition, the enzymatic approach yielded higher findings for creatinine and subsequently for MELD scores in patients who were liver transplant candidates. When the bilirubin concentration was above the 4 mg/dL threshold, there were differences between the approaches for both the creatinine and the MELD score (*p* values: 0.0001 and 0.027, respectively). The chemical Jaffe is a readily available and considerably cost-effective method for measuring creatinine. However, it is influenced by a variety of known and unknown interfering substances, and it should be applied cautiously when working with icteric samples. Alternate techniques such as the enzymatic method should be considered when the bilirubin level exceeds 4 mg/dL. Though this cut-off is instrument and kit-dependent, each laboratory is advised to have its cut-off for bilirubin interference.

## 1. Introduction

The Model for End-Stage Liver Disease (MELD) scoring system was introduced in the USA in 2002 and is used in many countries to prioritize liver allocation for most patients who require transplantation and to differentiate the severity of liver diseases. In fact, it is based on the “sickest first” principle. According to multiple studies, MELD can predict three-month mortality for patients on the liver transplant waiting list with an accuracy of about 80%. Pretransplant mortality was observed to increase exponentially rather than linearly with a change in the MELD score; as a result, changes of one or two points near the upper end of the MELD score are very clinically significant. Moreover, the MELD is a helpful clinical aid in a wide range of hepatic disease severity and variety. It incorporates commonly used laboratory tests, including the International Normalized Ratio (INR), serum creatinine, and serum total bilirubin. Unlike the objectivity of these three variables, the MELD score may be subject to some limitations based on how the parameters, especially creatinine, are measured [1–5].

Creatinine is measured using different automated methods, which include chemical Jaffe and enzymatic methods on automated analyzers, high-performance liquid chromatography (HPLC), and isotope dilution-mass spectrometry (IDMS). IDMS is the reference method of creatinine measurement, but it is not practical for routine usage [6–8].

The chemical Jaffe is one of the earliest methods for creatinine measurement, in which creatinine reacts with picrate under alkaline conditions to produce a yellow-red substance that is spectrophotometrically measured at a wavelength of 505 nm. It was first introduced in 1886 and is still in use today with some modifications due to its greater availability and cost-effectiveness [9–12]. However, major analytical problems are associated with the Jaffe reaction, particularly *those relating to positive and negative* interference by chromogens. More than 50 chromogenic interferents have been documented [13]. Glucose, uric acid, antibiotics, keto acids, bilirubin, and other chromogens interfere with creatinine measurement, and it may be measured higher or lower than the actual value. The original Jaffe method has undergone numerous modifications to reduce interference by such substances, with varying degrees of success [14, 15]. Although these modifications can correct interference from slow-reacting noncreatinine chromogens (glucose, acetone, and ascorbic acid), fast-reacting substances such as alpha-keto compounds and cephalosporin antibiotics give positive interference. In contrast, serum bilirubin negatively interferes with creatinine results and is a serious concern for clinical labs. Both conjugated and unconjugated bilirubin are disturbing factors as well as bilirubin breakdown products [3, 4, 10, 14].

Prior studies have demonstrated poor agreement and significant variation (low and high) between different creatinine measurement methods in specimens with high bilirubin concentration (icteric samples) and the MELD scores subsequently [4, 9, 10]. In a study by Evangelos Cholongitas et al., four different creatinine assays, including O'Leary modified Jaffe, compensated kinetic Jaffe, enzymatic, and standard kinetic Jaffe, were compared in patients with aberrant liver function tests. There was poor agreement between different methods, and increased variability in creatinine results and MELD scores occurred with increasing bilirubin concentrations [10]. Moreover, Carol Goulding et al. showed a lack of reproducibility of creatinine measurement and MELD scoring among four liver transplant units, and in two studies by Thorsten Kaiser et al., the Jaffe-based method showed greater creatinine levels than the enzymatic methods [4, 9, 16]. These discrepancies are worse with more severe jaundice and are sufficient to allow a patient to die while on the waiting list who may otherwise have received a transplant if his blood had been analyzed by a different method.

Similarly, the estimated glomerular filtration rate (GFR) calculation for chronic kidney disease (CKD) is another issue with the diversity of methods to measure serum creatinine. A patient's estimated GFR-based classification can vary significantly depending on small analytical changes in serum creatinine [17–24].

Since the current method for measuring creatinine (chemical Jaffe) is affected by high serum bilirubin, we conducted this study to compare the chemical Jaffe method with the more precise enzymatic method in icteric samples and assess the impact of various icteric levels on liver transplant allocation. Furthermore, we aimed to establish a trustworthy cut-off for bilirubin interference in the Jaffe method.

## 2. Materials and Methods

This cross-sectional study was conducted in the Clinical Chemistry Laboratory of Abu-Ali Sina Hospital, Shiraz, Iran, a transplantation center, from May 2022 to November 2022. The study was designed following the Declaration of Helsinki after obtaining approval from the Ethics Committee of Shiraz University of Medical Sciences (IR.SUMS.MED.REC.1400.105).

**Figure 1:**
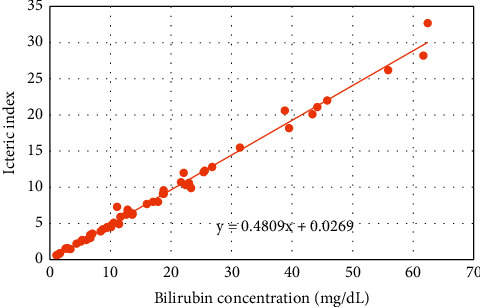
The relationship between the icteric index and total bilirubin level using 56 serum specimens with total bilirubin levels ranging from 1.07 to 62.4 mg/dL.

The icteric index was set up on an autoanalyzer for a simpler selection of icteric samples, using 56 serum samples with different levels of bilirubin, absorbance measurement at various specific bichromatic wavelength pairs (480 and 505 nm), and 0.9% sodium chloride as a reagent. The icteric index is a cost-effective, quick, and simple method for estimating hyperbilirubinemia [25, 26]. The relationship between total bilirubin level and icteric index is depicted in Figure 1.

Next, over a month, 400 residual serum samples from 356 individuals who were referred to the lab for various clinical issues were selected and categorized into four groups based on their icteric indices and total bilirubin levels, including nonicteric (bilirubin: ≤1.3 mg/dL), mild (bilirubin: 1.4 –4 mg/dL), moderate (bilirubin: 4.1–15 mg/dL), and severe (bilirubin: >15 mg/dL) icteric serum samples. Then, the specimens of all four groups were analyzed for creatinine using both chemical Jaffe and enzymatic methods, and the results were compared.

Concurrently, the specimens of patients who were candidates for liver transplantation (83 patients) were analyzed and interpreted similarly. All samples were stored at −20°C before analysis. Then, the MELD scores were calculated and compared in three groups, based on bilirubin level, with the formula, according to the guidelines of the United Network for Organ Sharing [27].

(1) $$\begin{array}{c} 10\times \left\{00.957\times \text{Loge}\left(\text{creatinine}\left[\frac{\mu \text{mol}}{L}\right]*A\right)+0.378\times \text{Loge}\left(\text{bilirubin}\left[\frac{\mu \text{mol}}{L}\right]*B\right.+1.120\times \text{Loge}\left(\text{INR}\right)+0.643\right\},\\ A=00.01131=\frac{\left(\text{creatinine}\left[\text{mg}/\text{dL}\right]\right)}{\left(\text{creatinine}\left[\mu \text{mol}/L\right]\right)},\\ B=00.05848=\frac{\left(\text{bilirubin}\left[\text{mg}/\text{dL}\right]\right)}{\left(\text{bilirubin}\left[\mu \text{mol}/L\right]\right)},\\ {}*\text{The upper limit of serum creatinine was capped at }4\;\frac{\text{mg}}{\text{dL}}. \end{array}$$

The measurements for creatinine were performed concurrently by the manufacturer's instructions using a DIRUI 1200 autoanalyzer after the two methods had been calibrated and quality control results had been confirmed. The reagents for the measurement of bilirubin and creatinine (Jaffe and enzymatic methods) were obtained from Biorex (Table 1). The INR was derived from prothrombin time (PT) measured using a Stago coagulation analyzer. None of the patients were taking either ascorbic acid or antibiotics. In addition, low-volume serum specimens and those with concurrent hemolysis and/or lipemia were excluded from the study.

## 3. Results

**Figure 2:**
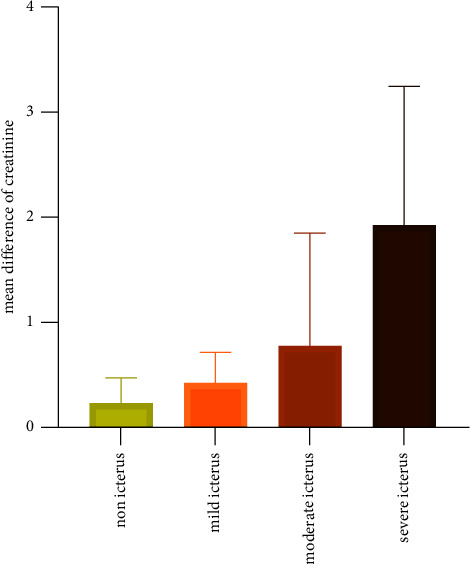
The mean differences of creatinine using Jaffe and enzymatic methods at various levels of icterus.

In nonicteric samples, there were no discernible differences between the Jaffe and enzymatic methods for measuring creatinine; however, in icteric samples, the enzymatic approach indicated a substantial increase (*p* value 0.0001), with a rising trend from the mild to severe icteric group (Table 2).  Figure 2 depicts the connection between the Jaffe and enzymatic approaches in these groupings.

**Figure 3:**
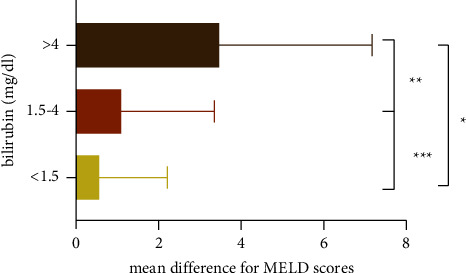
The mean differences of MELD scores between both methods. ^*∗*^*p* value: <0.0001, ^*∗∗*^*p* value: 0.027, ^*∗∗∗*^*p* value: 0.351.

Similar alterations in creatinine and MELD scores were found in 83 samples from liver transplant candidates during the second investigation (Table 3). The mean MELD score differences between the two approaches are shown in Figure 3.

## 4. Discussion

It is generally known that bilirubin negatively affects the Jaffe method's estimate of serum creatinine. The exact mechanism of bilirubin interference is not well known. However, bilirubin is converted to biliverdin under alkaline conditions, which results in a drop in absorbance at 510 nm (the absorbance peak of the creatinine picrate complex) and an increase at 630 nm (the absorbance peak of biliverdin), underestimating the concentration of creatinine. So, excess bilirubin results in a negative interference (lower creatinine values) that increases with increasing serum bilirubin concentrations and is typically found in the sickest patients with the greatest priority for liver transplantation [3, 4, 10, 14]. However, bilirubin interference in the Jaffe method appears to be more manufacturer-dependent, and few researchers have found positive interference when using compensated Jaffe methods [7, 28, 29].

This interference can be solved in several ways, including sample dilution, rate-blanking, the addition of oxidizing agents (ferricyanide), and deproteinization of the serum. The serum dilution and rate-blanking methods are currently applied to some reagents available, with varying degrees of success. However, pretreatment by deproteinization of patients' serum and oxidizing agents cannot be routinely utilized because it cannot be automated and requires manual operation [13, 14, 30].

Alternatively, creatinine concentrations can be measured enzymatically. Several enzymes, such as creatinine amidohydrolase and creatinine kinase, can convert creatinine to creatine with a subsequent absorbance change at 340 nm. This method has been reported to be more resistant to bilirubin interference and improve the specificity of the measurement. According to previous studies and the manufacturer's specifications, the enzymatic approach appears more appropriate as a routine laboratory technique for measuring icteric serum creatinine [31]. However, it is considerably more expensive than the kinetic Jaffe method [7, 9, 32, 33].

In this study, the effectiveness of the Jaffe and enzymatic methods in icteric samples was compared at various icterus levels. The creatinine concentrations showed higher results using the enzymatic method than the Jaffe method, and as bilirubin levels rose, the mean differences in creatinine widened. Furthermore, the enzymatic method produced higher results for creatinine and MELD scores in patients who were candidates for liver transplantation. The differences between the methods for creatinine and MELD scores were significant when bilirubin concentration crossed the border of 4 mg/dL, which is consistent with the manufacturers' claim regarding the degree of bilirubin interference. Likewise, various limits for bilirubin interference have been established by previous research (i.e., 25 mg/dL) using different reagents and analyzers [1].

The lower creatinine and MELD scores by the Jaffe method will cause patients to be misplaced on the waiting list and delay receiving liver transplants. These findings restrict the application of the Jaffe method for creatinine measurement in icteric samples.

In a laboratory, a test's cost-effectiveness is just as essential as its accuracy. The cost-effectiveness of a test is significant when it is sensitive and specific enough to make a diagnosis [34, 35]. As a result, in our laboratory, the enzymatic approach should be reserved just for instances where the bilirubin level is greater than 4 mg/dL.

## 5. Conclusion

The chemical Jaffe is a readily available and considerably cost-effective method for measuring creatinine. However, it is influenced by a variety of known and unknown interfering substances, and it should be applied cautiously when working with icteric samples, and alternate techniques such as the enzymatic method should be considered when the bilirubin level exceeds 4 mg/dL. Though this cut-off is instrument and kit dependent, each laboratory is advised to have its cut-off for bilirubin interference.

## Figures and Tables

**Table 1:** Characteristics of methods used in the study.

Analytes	Method	Reagent	Wavelength (nm)	Analytical sensitivity (mg/dL)	Linearity limit (mg/dL)	Limit of icterus interference, bilirubin (mg/dL)
Creatinine	Jaffe	BIOREX	500	0.2	20	4
Creatinine	Enzymatic (creatinine deiminase)	BIOREX	340	0.2	20	15
Bilirubin	Jendrassik- Grof	BIOREX	546	0.1	25	—

IBM SPSS (version 25.0) was used to analyze all the data. Quantitative variables were expressed as mean ± SD and/or median (range). Significance testing was 2-sided and set to less than 0.05. The Wilcoxon signed-rank test was used for a nonparametric comparison between paired *Cr* values and paired MELD scores. The Mann–Whitney *U* test was used to determine how the mean values differed.

**Table 2:** Comparison of 4 groups regarding Jaffe and enzymatic creatinine results.

Groups	Number of samples	Degree of icterus	Total bilirubin (mg/dL) (mean ± SD)	Creatinine Jaffe (mg/dL) (mean ± SD)	Creatinine enzymatic (mg/dL) (mean ± SD)	*P* value
1	100	Nonicteric	0.64 ± 0.24	1.71 ± 1.41	1.79 ± 1.38	0.237
2	100	Mild icteric	2.2 ± 0.77	1.39 ± 1.06	1.81 ± 1.14	<0.0001
3	100	Moderate icteric	8.3 ± 5.34	1.2 ± 0.95	1.91 ± 1.20	<0.0001
4	100	Severe icteric	30.8 ± 17.31	1.33 ± 1.04	3.28 ± 1.89	<0.0001

**Table 3:** Data from specimens of liver transplantation candidates.

Total bilirubin (mg/dL)	Number of cases	Bilirubin range (mean) (mg/dL)	Mean difference of creatinine
<1.5	34	0.12–1.48 (0.75)	0.08 ± 0.42
1.5–4	21	1.51–3.82 (2.37)	0.10 ± 0.28
>4	28	4.53–58.4 (17.40)	0.78 ± 0.80
Total	83	0.12–58.4 (6.78)	0.32 ± 0.64

## Data Availability

The data used to support the findings of this study are included within the article.
